# Self-regulated and socially shared regulation of learning in adaptive collaborative educational environments: a scoping review

**DOI:** 10.1186/s12909-026-09355-9

**Published:** 2026-06-15

**Authors:** Faiza Ikram, Rahila Yasmeen, Humaira Fayyaz

**Affiliations:** 1 Department of Physiology, Nishtar Medical University, Multan, Pakistan; 2 Riphah Academy of Research and Education, Riphah International University, Islamabad, Pakistan; 3 Faculty of Health & Medical Sciences, Riphah International University, Islamabad, Pakistan

**Keywords:** Self-regulated learning, Socially shared regulation, Collaborative learning, Adaptive learning, Health professions education

## Abstract

**Background:**

Collaborative learning is central to higher education, yet its effectiveness depends on learners’ ability to regulate learning both individually and collectively. While self-regulated learning (SRL) and socially shared regulation of learning (SSRL) are recognised as complementary processes, their interaction within adaptive collaborative learning environments remains insufficiently synthesised. The objective of this scoping review was to map and synthesise existing literature describing the components, processes, and adaptive strategies underpinning the interplay between SRL and SSRL in collaborative learning contexts in higher education, and to identify the current evidentiary base within high-stakes fields like health professions education.

**Methods:**

A scoping review was conducted following the Arksey and O’Malley framework and reported in accordance with PRISMA-ScR guidelines. Searches were performed across PubMed/MEDLINE, Google Scholar, ScienceDirect, Semantic Scholar, and Taylor & Francis for studies published between 2004 and 2024. Eligible studies addressed SRL, SSRL, collaborative learning, and/or adaptive learning in higher or health professions education. Data were systematically charted and analysed using a hybrid inductive-deductive thematic analysis.

**Results:**

A total of 31 studies met the inclusion criteria, including 14 empirical, 12 conceptual/theoretical, 4 review-based contributions and one bibliometric analysis. Regulation in collaborative learning was found to be dynamic, involving continuous shifts between individual and shared processes across affective, cognitive, metacognitive, and motivational domains. Recurrent challenges included emotional strain, misaligned goals, invisible reasoning, uneven participation, and coordination breakdowns. Adaptive learning supported regulatory processes through personalised feedback, scaffolding, dashboards, and prompts. However, these systems largely operated in parallel, addressing either individual regulation or group-level processes, with limited emphasis on harmonising SRL and SSRL. Empirical evidence from health professions education was limited.

**Conclusions:**

Effective collaborative learning depends on dynamic coordination between self- and socially shared regulation. Although adaptive learning environments show promise, current designs provide limited support for coordinating individual and collective regulatory processes. Notably, empirical research applying these frameworks within higher education is critically scarce, particularly in health professions education. This highlights a major gap for future theory-informed adaptive designs that must harmonise individual autonomy with shared accountability.

**Supplementary Information:**

The online version contains supplementary material available at 10.1186/s12909-026-09355-9.

## Introduction

Collaborative learning approaches, such as case-based learning, problem-based learning, team-based learning, and simulation-based education are increasingly emphasized across higher education to prepare students for complex, interdependent workplaces [4]. Despite this emphasis, the full potential of collective learning outcomes in collaborative settings remains inadequately realized [7, 12]. One key reason for this limitation lies in the regulatory demands inherent in collaborative learning [7, 12].

To navigate these environments effectively, learner must engage in multiple, overlapping forms of regulation. At the individual level, learning is guided by self-regulated learning (SRL), which encompasses cognitive, metacognitive, behavioral, and emotional processes that support goal setting, performance, and reflection [34, 35]. However, when learning occurs in groups, regulation expands beyond the individual. This transition frequently involves co-regulation (CoRL), an asymmetric process in which one learner, facilitator, or learning environment temporarily scaffolds another’s regulatory processes [2]. While CoRL helps maintain momentum, highly effective collaborative learning ultimately requires a shift toward socially shared regulation of learning (SSRL) [2, 6]. SSRL is an evenly distributed, reciprocal and collectively negotiated process where learners coordinate strategies, co-construct shared goals, manage interpersonal dynamics, and respond to collective cognitive, motivational, and socio-emotional challenges at a metacognitive level [12].

Learners vary widely in their regulatory capacities, including cognitive and metacognitive skills, motivation, emotional states, pacing, learning preferences, prior knowledge, and interpersonal relationships [12, 34]. When these differences create misalignment between individual autonomy and group-level regulatory demands, collaborative learning may fail [7, 12]. Research indicates a reciprocal relationship between self-regulated learning (SRL) and socially shared regulation of learning (SSRL) [34, 43]. Individuals contribute their self-regulatory skills to the groups, which are negotiated through social interaction, while engaging in SSRL concurrently shapes individual SRL by exposing learners to diverse strategies [40, 44]. This dynamic interplay highlights the need for learning environments that facilitate flexible transition between individual and shared regulation rather than privileging one form over the other.

Adaptive learning environments have demonstrated potential in supporting this dynamic interplay [22, 40]. At the individual level, adaptive systems promote autonomy by providing personalized feedback and progress monitoring [22]. In collaborative contexts, adaptive learning can support shared planning and shared metacognition by making learning processes visible [40, 47]. In this sense, adaptive learning environments may act not only as personalization tools but also as mediating systems that shape how individual and group regulation processes unfold [21, 40].

Despite this potential, empirical efforts to harmonize these processes within adaptive collaborative settings remain limited [34, 40]. A major contributing factor is the lack of a coherent conceptual and theoretical understanding of how SRL and SSRL interact within adaptive systems [34, 40]. Recent reviews, such as Sharma et al., have comprehensively mapped the technological architectures of adaptive learning environments that foster regulation [40]. There remains a critical need to synthesise the process-level interplay and map specific regulatory challenges that learners face during day-to-day collaboration.

Furthermore, while these regulatory frameworks are extensively studied in general higher education and computer-supported collaborative learning (CSCL), the application of these regulatory models within the high-stakes, hierarchical structures of clinical training remains under-explored. Health professions education represents a uniquely high-stakes collaborative environment characterized by hierarchical team structures, patient safety concerns, and workplace-based learning, where failures in shared regulation carry significant consequences [4].

To address these gaps, the objective of this scoping review was to map and synthesize existing literature describing the components, processes, and adaptive strategies underpinning the interplay between self-regulated learning and socially shared regulation of learning within collaborative learning contexts in higher education. Additionally, this review seeks to critically evaluate the extent to which this evidentiary base has been established within health professions education, outlining critical gaps for future research.

## Methodology

A scoping review methodology was selected because the interaction between self-regulated learning (SRL), socially shared regulation of learning (SSRL), and adaptive learning in collaborative settings is complex and insufficiently synthesized. Scoping reviews are useful for mapping broad bodies of literature, clarifying conceptual boundaries, and identifying gaps in emerging fields [33]. This review followed the Arksey and O’Malley framework with enhancements described by Levac et al., [25]. The reporting adhered to PRISMA-ScR guidelines, with relevant elements from PRISMA-2020 where appropriate [31, 42]. The aim was to explore how SRL and SSRL interact within collaborative learning, and which adaptive strategies are described to harmonize these regulations.

The primary research question guided the review: “What are the key components, processes, and adaptive strategies described in existing literature on Self-Regulated Learning (SRL), Socially Shared Regulation of Learning (SSRL), and adaptive learning in collaborative higher educational settings?” This question was refined after an initial exploration of the field and included the identification of conceptual ambiguities surrounding the interplay of regulatory levels. Additionally, a secondary objective was established to address the applied context of the review, to determine the extent to which this evidentiary base has been established within health professions education.

### Search strategy

A structured methodological search strategy was developed to identify relevant literature. The initial search strategy was developed in PubMed/MEDLINE using Medical Subject Headings (MeSH), controlled vocabulary, free-text terms, truncation, and Boolean operators. Search terms covered core concepts such as self-regulated learning, socially shared regulation, co-regulation, collaborative learning, adaptive learning environments, higher education, and medical education. Search strings were iteratively refined using synonyms and controlled vocabulary to maximize both sensitivity and specificity.

The finalized search strategy was adapted for Google Scholar, ScienceDirect, Semantic Scholar, and Taylor & Francis. These databases were selected for their strong coverage of education and medical education research. To broaden the scope, Google Scholar was included to capture literature that may not appear in traditional indexed databases. Additional searches included reference list screening, citation tracking, snowballing, and expert consultation.

The selected databases (PubMed, ScienceDirect, Taylor & Francis, and Semantic Scholar) were chosen due to their comprehensive indexing of core journals in the Learning Sciences and Computer-Supported Collaborative Learning (CSCL). While ERIC and PsycInfo are valuable, the high degree of overlap between these platforms and our selected databases, combined with the use of Google Scholar and citation tracking to capture outliers, ensured a robust capture of the core SRL/SSRL literature.

Grey literature was screened selectively to capture emerging conceptual and empirical work not yet indexed in peer-reviewed journals. Editorials, commentaries, letters, and conference abstracts were excluded; however, theses, dissertations, and preprints were included when they met the relevance criteria and contributed meaningfully to the scope of the review. The search was conducted over four months (February–May 2025) and covered studies published from 2004 to 2024 (including advance online publications), reflecting the developmental trajectory of regulatory learning theories.

### Search terms

The search employed predefined keywords, MeSH terms, and synonyms, Table 1. Boolean operators “OR” broadened individual concept groups, while “AND” linked major concepts. Variants such as “self-control,” “team learning,” “personalized learning,” and “intelligent tutor” were included to capture conceptual breadth. An example of the combined search string was:

- (Self-regulat* OR emotional regulation).
- AND (social* shared regulat* OR collaborat* learn* OR co-regulat* OR group learn*).
- AND (adapt* learn* OR personaliz* learn* OR intelligent tutor*).
- AND (education OR teaching OR medical education OR higher education).

**Table 1:** Keywords and controlled vocabulary used in the literature search

Keywords	Synonyms
Self-regulation	Self-control, Emotional regulation
Socially shared regulation	Shared regulation, co-regulation, shared decision-making, social learning
Collaborative learning	Collective learning, team learning, group learning
Adaptive learning	Adaptive learning environment, personalized learning, individualized learning, adaptive education, intelligent tutor
Education	Teaching, learning, educational models, medical education, higher education, instructional models

Complete search groupings appear in Annexure 1. Furthermore, the primary search string relied on root terms for socially shared regulation. During the screening process, however, studies investigating ‘shared metacognition’, conceptually defined by Iiskala et al., as the collective emergence of monitoring, were treated as synonymous with SSRL to ensure this critical group-level construct was captured [9].

### Study selection

All retrieved citations were imported into Zotero, where duplicates were removed through automated and manual checks. Two reviewers independently screened the retrieved articles in three stages: titles, abstracts, and full-text review. Disagreements were resolved through discussion or consultation with a third reviewer. Studies were included if they were published in English, peer-reviewed or qualifying grey literature (theses or preprints), and focused on SRL, SSRL, co-regulation, collaborative learning, or adaptive learning within higher education or health professions education settings. To ensure population validity and align with the unique regulatory demands of adult learners, studies focusing on primary or secondary (K-12) populations were explicitly excluded. Studies addressing only individual regulatory processes without collaborative components, or grey literature such as editorials and commentaries, were excluded (Table 2).

**Table 2:** Inclusion and exclusion criteria for study selection

Category	Criteria
Inclusion Criteria	• Studies published between 2004–2024 • Empirical studies, theoretical papers, review articles, or meta-analyses, relevant thesis or preprints focusing on SRL, SSRL, collaborative learning, or adaptive learning • Published in English • Conducted in educational settings, with emphasis on healthcare and higher education • Quantitative, qualitative, or mixed-methods research designs
Exclusion Criteria	• Grey literature not peer-reviewed, such as opinion pieces, editorials, commentaries, conference abstracts, letters to editors, and short communications • Studies focused only on individual learning without collaborative or regulatory components • Studies conducted in K-12 settings (primary, middle, or high school classrooms

### Data extraction

A structured data charting form was developed and piloted using three articles that represented diverse methodological designs. Once finalized, two reviewers independently extracted data from all included studies. Extracted information included author details, publication year, research setting, conceptual or theoretical model referenced, regulatory processes addressed, descriptions of SRL–SSRL interactions, challenges highlighted, and adaptive strategies proposed (Annexure 2).

To ensure consistency during screening and extraction, we operationalized the three primary constructs based on Hadwin et al., [6]: Self-Regulated Learning (SRL) as individual-level deliberate task engagement; Co-regulation (CoRL) as the asymmetric, transitional scaffolding of one person’s regulation by another; and Socially Shared Regulation (SSRL) as the symmetric, collective negotiation of a shared task. Discrepancies in extraction were resolved through discussion following the same procedure used during screening.

Geographical distribution was determined based on the reported study setting and participant context. If not explicitly stated, location was inferred from the described educational setting and institutional context only when all authors were affiliated with the same institution and no indication of multi-country data collection was present.

### Quality appraisal

Although optional in scoping reviews, methodological appraisal was performed to enhance rigour. The QualSyst tool [23] was applied. Quantitative/mixed-methods studies were evaluated on a 28-point scale and qualitative studies on a 20-point scale. Criteria were scored 0, 1, or 2. Studies scoring ≥ 60% were considered sufficiently robust for synthesis.

### Data analysis

The included studies were analysed using Braun and Clarke’s six-step thematic analysis [3]. Two reviewers independently familiarised themselves with the data and generated initial codes using a hybrid inductive-deductive approach. Theoretical constructs of SRL/SSRL guided initial categorisation (deductive), while specific regulatory challenges and adaptive strategies emerged directly from the data (inductive).

These codes were then clustered into potential themes, which were refined through iterative mapping and review. To ensure interpretive rigour, the primary reviewers engaged in regular consensus-seeking meetings to resolve any discrepancies in coding or theme development. Given the conceptual heterogeneity of the data, meta-analysis was not feasible. This methodological approach enabled comprehensive mapping of the conceptual foundations, regulatory challenges, and adaptive mechanisms described across SRL, SSRL, and adaptive learning literature.

## Results

### Search results and study selection

Initial database searches yielded large result sets for individual constructs. When combined using Boolean operators, the finalised search string yielded 1,148 records across all databases. An additional seven records were identified through reference screening and citation tracking.

**Fig. 1:**
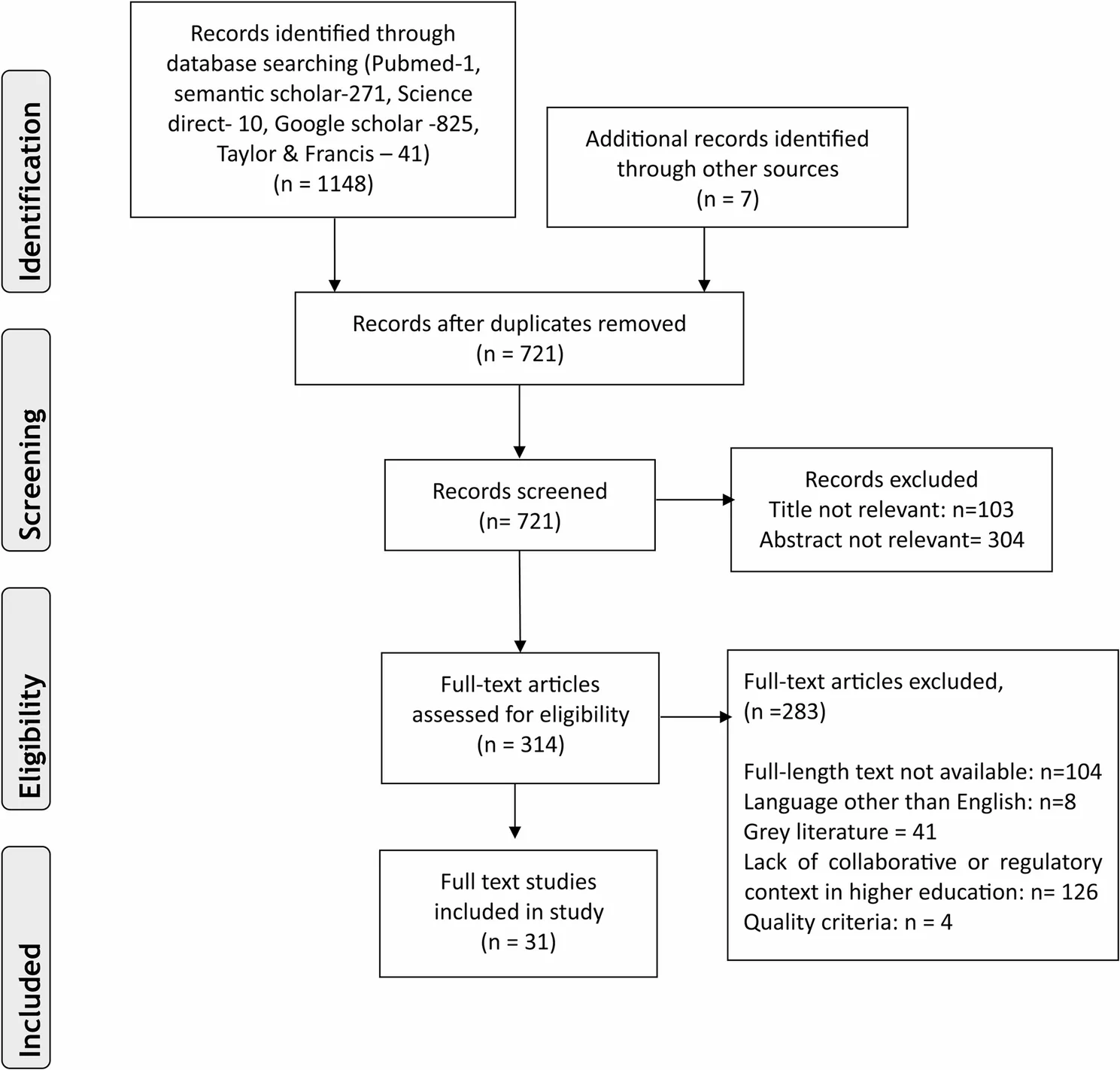
PRISMA flow chart diagram for selection of studies

After removing duplicates, 721 records proceeded to title and abstract screening. These were evaluated against predefined inclusion and exclusion criteria. A total of 58 full-text articles were assessed for eligibility, and 31 studies met all criteria and were included in the final review. The PRISMA flow diagram summarizing the identification, screening, and inclusion process is presented in Fig. 1.

### Study designs, context and participant characteristics

The 31 included studies represent a diverse body of work spanning self-regulated learning (SRL), co-regulation, socially shared regulation of learning (SSRL), collaborative learning, and adaptive learning systems. Study designs included quantitative (*n* = 2), qualitative (*n* = 4), mixed methods (*n* = 8) empirical studies, alongside non-empirical contributions, including conceptual/theoretical papers (*n* = 11), literature reviews (*n* = 4; three systematic and one narrative), and one bibliometric analysis. This distribution reflects both methodological breadth and the conceptual richness of the field.

Studies covered a wide range of educational settings, with higher education as the dominant context. Most empirical work took place in university-level courses across chemistry, mathematics, English, teacher education, computer science, online programming, multimedia design, Massive Open Online Course (MOOC) environments, and health professions education. Graduate-level computer-supported collaborative learning (CSCL) courses also appeared frequently. A sizeable group of contributions were conceptual/theoretical without focusing on a specific learner population.

Only two studies focused directly on health professions education, and both were conceptual discussions framing SRL, co-regulation, and SSRL within the context of healthcare teamwork and collaborative clinical practice. No empirical studies examined SRL or SSRL processes among medical, nursing, dentistry, pharmacy, or allied health students. This highlights a clear gap in applied research within healthcare education, despite strong conceptual grounding. Annexure 3 summarises the characteristics of included studies, including study design, context, key concepts, major findings, and extracted themes.

### Publication years distribution

**Fig. 2:**
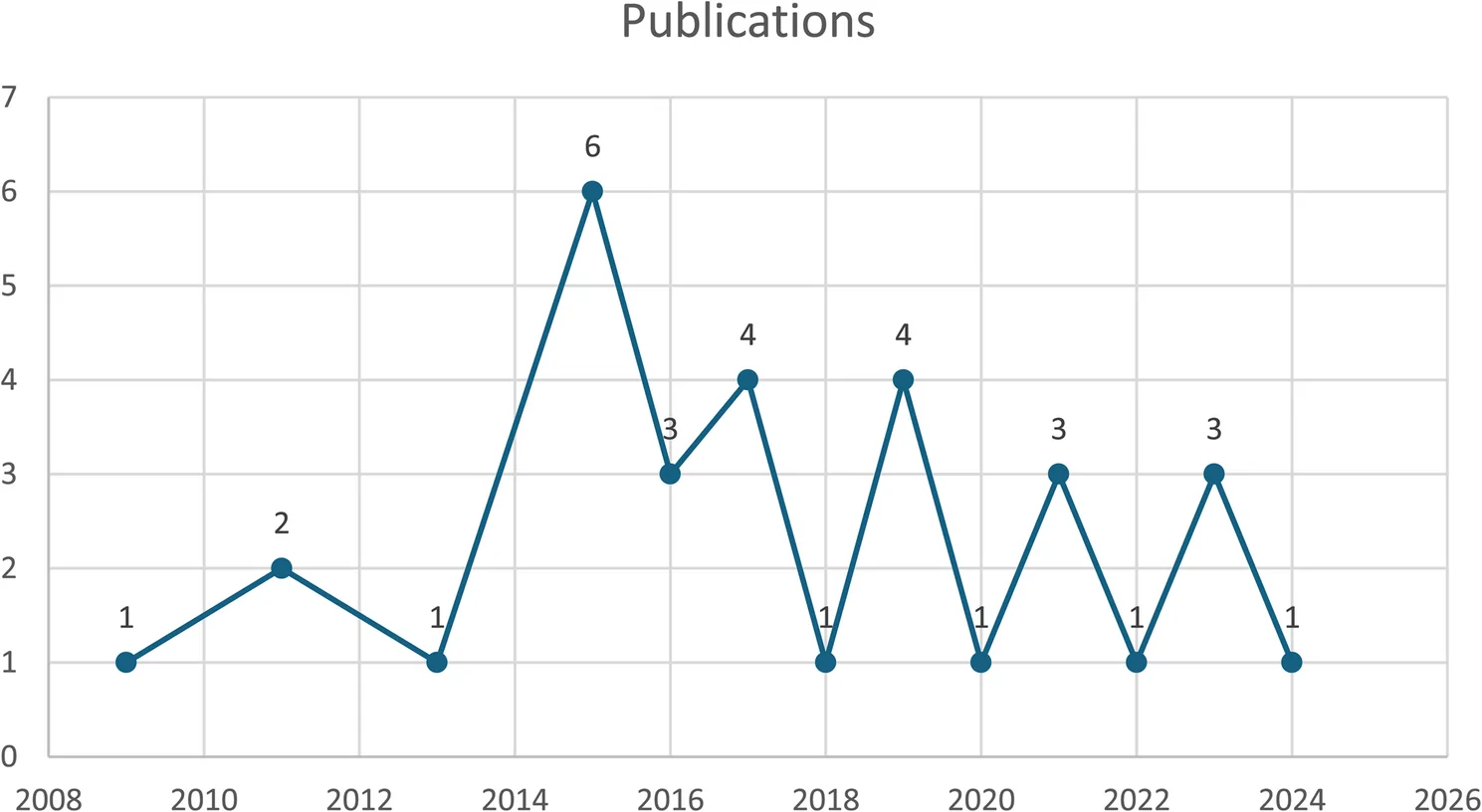
Year-wise distribution of included studies (*n* = 33; 2004–2024)

The 31 included studies span a 15-year period from 2009 to 2024, showing a clear increase in research activity related to SRL, SSRL, and adaptive regulation in collaborative learning (Fig. 2). Three distinct phases were identifiable based on publication trends and methodological developments:

#### 2009–2015: Foundational phase

This early period included influential conceptual and theoretical contributions, such as Hadwin & Oshige [7] and Järvelä & Hadwin [11], alongside early empirical mixed-methods work [16], [16]. These studies established foundational models of SRL and evolving perspectives on co-regulation and socially shared regulation. Methodological papers published during this phase laid groundwork for the process-oriented approaches that followed [16].

#### 2016–2020: Growth phase

Research increased markedly during this period, with expansion in empirical and mixed-methods studies using sequential analysis, video-based micro-analytics, trace data, and epistemic network analysis. Notable contributions include Järvelä et al., [15], Isohätälä et al., [10], Malmberg et al., [28]d rvenoja et al., [19]. These studies advanced understanding of regulation during computer-supported collaborative learning and provided methodological refinements for capturing the temporal and social dynamics of regulation.

#### 2021–2024: Emerging AI-supported phase

The most recent stage reflects a shift toward adaptive, computational, and AI-enhanced regulation research, including multimodal analytics, physiological synchrony, intelligent scaffolding, and randomized trials [26, 32, 40]. Reviews published during this phase synthesized technology-enhanced regulatory processes and examined the role of adaptive learning in detecting and supporting regulation.

#### Overall trends

More than half of all included studies were published between 2017 and 2024, indicating a growing research emphasis on collaborative regulation, multimodal data, and adaptive learning technologies. The steady upward trajectory suggests increasing theoretical maturation and methodological sophistication in examining regulation within collaborative and adaptive learning contexts.

### Geographic distribution of studies

Most empirical studies were conducted in Finland, particularly in computer-supported collaborative learning contexts. Additional studies were conducted in China, the United States, Portugal, Germany, Australia, and Japan. A small number of studies were conducted in online or MOOC-based environments. Conceptual papers and systematic reviews were excluded from the geographical analysis.

### Evidence mapping of included studies

The included studies were mapped onto four major regulatory domains aligned with the review objectives: self-regulated learning (SRL), socially shared regulation of learning (SSRL), collaborative regulatory dynamics, and adaptive regulation. Because the reviewed literature frequently investigated overlapping regulatory processes (e.g., a single study exploring both SSRL and adaptive technologies), the studies were mapped to multiple relevant domains. Therefore, the categorical counts presented below are not mutually exclusive and sum to more than the total 31 included studies.

#### Self-Regulated Learning (SRL)

Eleven studies examined SRL either as the main focus or as part of a broader regulatory model. These studies described SRL as a cyclical process involving planning, monitoring, strategy use, persistence, emotional management, and self-reflection [7, 8, 45, 47]. Conceptual and theoretical work drew on foundational frameworks such as Zimmerman’s cyclic model, Winne and Hadwin’s COPES architecture, and Pintrich’s self-regulation framework, often synthesised in model reviews of SRL [11, 17, 18, 34, 39]. Empirical studies explored how learners applied SRL in online courses, adaptive platforms, and cognitively demanding collaborative tasks, using log-trace data, adaptive assessments, and questionnaire measures to capture learners’ regulatory actions [8, 24, 47]. Together, these studies consistently framed SRL as an individual, proactive process shaped by cognitive and affective factors and increasingly supported through digital and adaptive tools.

#### Socially Shared Regulation of Learning (SSRL)

Twenty-six studies focused on SSRL either as a primary focus or alongside SRL. These studies showed how groups engage in shared planning, monitoring, evaluation, and strategic adjustment during collaborative tasks [10, 11, 13, 28, 36]. Conceptual papers traced the evolution of SSRL theory from the Winne–Hadwin model toward frameworks that integrate emotional and motivational regulation. Across empirical studies, high-performing groups showed explicit negotiation, shared goal alignment, and coordinated monitoring, whereas low-performing groups demonstrated fragmented or reactive regulatory patterns and more frequent socio-emotional challenges [16, 19, 20, 26]. Overall, SSRL appeared strongly linked to group emotional climate and the quality of interaction.

#### Collaborative learning regulatory dynamics

Twenty-six studies explored how SRL, co-regulation, and SSRL operate together during collaborative tasks. These studies conceptualized regulation as a dynamic, layered process that shifts across individuals, dyads, and groups depending on task demands [7, 11, 39]. Common regulatory challenges included unequal participation, misaligned task understanding, coordination difficulties, and socio-emotional tensions such as frustration, withdrawal, or tense group climate [20, 26, 29]. Process-oriented methods such as temporal analysis, process discovery, and network analysis were frequently used to trace how regulatory behaviours emerged, escalated, or broke down related to collective performance [2, 28, 48]. Collectively, these findings showed the complexity of collaborative regulation and underscored the need for structured supports that help groups coordinate cognitive, motivational, emotional, and behavioural processes.

#### Adaptive regulation and technology-enhanced supports

Sixteen studies examined adaptive learning systems or digital tools designed to support regulation. To systematically conceptualize these supports, Yen et al., proposed a comprehensive framework for Self-Regulated Digital Learning (SRDL). Their framework identifies core technological affordances that directly facilitate the transition of regulatory skills into digital environments [47]. These studies described a wide range of adaptive mechanisms, including intelligent tutoring systems, dynamic content sequencing, multimodal sensing, real-time feedback, and group-aware dashboards [1, 21, 27, 30]. Several SRL-focused studies showed how adaptive systems modelled learner states and adjusted task difficulty, hints, or feedback to support planning, monitoring, and strategy use [8, 47]. Other work used adaptive prompts, emotion–motivation dashboards, group awareness tools, or multimodal trigger-based designs to support co-regulation and SSRL during collaboration [14, 19].

A subset of ten dual-focus studies explicitly linked adaptive mechanisms to regulation by showing how adaptive prompts, alerts, and dynamic scaffolds mediate transitions from individual SRL to co-regulation and SSRL [14, 32, 40, 41, 48]. While adaptive systems demonstrated strong support for individual personalization, far fewer studies offered robust solutions for group-level regulation. This highlights collective monitoring, shared emotion–motivation regulation, and coordinated task adaptation as key areas for future work [27, 38, 40].

### Thematic synthesis

The thematic analysis generated two major outputs:

- Regulatory conflicts between SRL and SSRL, and.
- Adaptive strategies used across domains.

#### Regulatory conflicts between SRL and SSRL

Across cognitive, metacognitive, emotional/affective, and motivational domains, studies described tensions such as mismatched goals, inconsistent monitoring strategies, variable emotional or motivational states, uneven task ownership, and technological or environmental disruptions, detailed in Table 3 and Annexure 4. It is important to note that the pairings of regulatory challenges with specific adaptive strategies presented in these summaries are conceptually synthesized mappings derived from thematic proximity in the literature, rather than direct, empirically tested 1:1 causal mechanisms. These regulatory challenges are organized into four domains: emotional–affective, cognitive, metacognitive conflict and motivational regulation.

**Table 3:** Thematic Synthesis of Included Studies (*n* = 31)

	Regulatory Challenges	Adaptive Strategies
Affective/Emotional	• Emotional Autonomy vs. Group Influence • Emotional Disconnect in Group Dynamics • Unregulated Affective Traits • Behavioural Style Clashes in Collaboration • Unequal Engagement & Task Ownership • Environmental & Attention Regulation Gaps	• Structured Emotional Regulation in Collaborative Settings • Socioemotional Awareness & Climate Tools • Emotionally Adaptive Group Structuring • External Behavioural Scaffolds • Equity-Based Behavioural Scaffolding • Task & Attention Management Tools
Cognitive	• Prior Knowledge & Cognitive Diversity Mismatch • Isolation from Over-Personalized Learning • Alignment & Visibility of Cognitive Processing • Disruptive Learning Design • Cognitive Load • Autonomy–Collaboration Balance	• Adaptive Group Formation & Matching • Modality & Content Adaptation for Cognitive Balance • Harmonization & Visualization of Cognitive Strategies • Cognitive Flexibility Tools • Task Complexity Matching & Chunking
Metacognitive	• Misaligned Monitoring & Regulation Skills • Overscripting vs. Underscripting • Misaligned Feedback & Scaffolds • Goal Misalignment • Inflexible Peer Interaction Models	• Shared Monitoring & Visualization Tools • Adaptive Scripting & Support Fading • Feedback for Regulation Adaptation • Goal Alignment & Tracking Systems • Reflection & Debriefing Integration
Motivational	• Goal Misalignment in Motivation • Variability in Engagement & Effort • Role Ambiguity • Autonomy vs. External Motivation Control • Lack of Shared Success Structures	• Goal Alignment & Motivation Mapping • Adaptive Feedback & Nudging Systems • Role-Based Motivation Design • Positive Interdependence Frameworks • Motivation-Driven Environment Design

*Challenge-strategy pairs across domains represent conceptual thematic links from the literature, not empirically tested 1:1 causal mechanisms

#### Adaptive strategies identified in the literature

Studies also described multiple adaptive mechanisms designed to address regulatory barriers. These strategies include adaptive feedback loops, dynamic scaffolding, shared monitoring systems, affect-responsive supports, adaptive task sequencing, and collaborative analytics. Full mappings and examples appear in Annexure 4. Below is the theme-level narrative for each regulatory domain:

##### Emotional / affective regulation

Studies highlighted the need to manage frustration, conflict, confusion, and affective strain during collaborative tasks [16, 26]. Emotional regulation stabilized group climate, supported positive engagement, and facilitated conflict resolution [10, 29]. Multimodal studies using physiological sensors demonstrated that group members’ affective states often aligned during complex tasks [29]. Supports included reflective prompts, scripted routines, and real-time alerts when disengagement or confusion persisted [14, 19]. These mechanisms helped groups maintain focus and regain engagement when emotional demands increased.

##### Cognitive regulation

Studies described goal setting, task planning, strategy selection, and progress monitoring as central cognitive processes in collaborative tasks [7]. Trace data and process-mining studies [24, 28] showed that groups often shifted from fragmented individual strategies toward more coherent shared strategies as collaboration progressed. Low-tech supports such as structured prompts and task templates assisted groups in clarifying goals and distributing responsibility [14]. In contrast, high-tech systems such as ALEKS adapted content difficulty and delivered performance-based hints to support cognitive regulation [8]. Cognitive regulation was most prominent during early task phases when groups negotiated problem understanding and task demands [28].

##### Metacognitive regulation

Metacognitive regulation involves planning, monitoring, and evaluating learning processes [7, 34]. Sequential analyses indicated that metacognitive monitoring frequently triggered transitions from individual regulation to socially shared regulation during collaborative tasks [28, 48]. Regulation emerged more consistently when environments provided cues to highlight knowledge gaps or coordination breakdowns [8]. The adaptive techniques range from reflective questions and visual planning tools to adaptive dashboards and AI-generated alerts indicating stagnation or unequal participation [19]. Furthermore, embedding features such as the visualization of procedures and integrated machine feedback directly into the interface is critical for closing the loop between individual monitoring and shared collective evaluation [47]. Observational data showed that metacognitive prompts often initiated collective sense-making rather than isolated self-reflection [10, 29].

##### Motivational regulation

Motivational regulation involved managing effort, persistence, task value, and declines in motivation during demanding tasks [7, 16]. High-performing groups expressed stronger task commitment, mutual encouragement, and distributed responsibility [24, 26]. Support strategies included low-tech approaches such as assigned team roles, progress checklists, and milestone-based task structures, which helped sustain engagement and accountability [14, 38]. In technology-supported environments, adaptive features such as system-generated encouragement prompts, progress visualizations, and group awareness indicators supported motivation regulation by making participation visible at the group level [19]. Motivational regulation was frequently intertwined with cognitive and metacognitive strategies during challenging phases of tasks [20, 28].

### Quality appraisal

A total of 14 empirical studies were appraised for quality assessment. Qualitative studies were appraised using the 10-item QUALSYST tool, and quantitative/mixed-methods studies using the 14-item version. Conceptual papers, methodological papers, design frameworks, systematic reviews, and bibliometric analyses were not appraised, in accordance with QUALSYST guidelines.

Quality scores ranged from 64% to 95%, detailed in annexure 3. Quantitative studies generally scored high on clarity of objectives, measurement robustness, and analytic methods. Lower scores were seen for issues related to sampling procedures or incomplete reporting of variance estimates.

Qualitative studies performed well on design clarity, data collection description, and analytic alignment, though some lacked depth in addressing researcher positioning or verification strategies. Mixed-methods studies displayed varied quality; those integrating trace data with video or survey analysis often reported strong methodological justification but were limited by incomplete reporting on sampling decisions or confounding control.

### Summary of evidence

Overall, the evidence shows that self-regulated learning and socially shared regulation of learning are closely interconnected, with frequent tensions emerging during collaborative tasks across cognitive, metacognitive, affective, and motivational domains [2, 7, 39]. Adaptive learning systems show potential in supporting regulation, ranging from low-tech structuring tools to AI-supported scaffolds [8, 14]. However, most systems primarily emphasised individual-level personalization, with limited support for group-level and socially shared regulation [27, 30, 36, 38].

## Discussion

This scoping review synthesised theoretical, conceptual and empirical literature to examine how self-regulated learning (SRL) and socially shared regulation of learning (SSRL) interact within collaborative learning environments, and how adaptive strategies support this interplay. The findings demonstrate that regulation in collaborative learning is inherently dynamic, involving continuous shifts between individual and shared processes across affective, cognitive, metacognitive, and motivational domains. Despite growing conceptual clarity, the literature remains fragmented, as adaptive learning designs often privilege either individual or group regulation rather than supporting both in a coordinated manner.

### Affective and emotional regulation

Across studies, affective regulation emerged as a critical yet vulnerable component of collaborative learning. Emotional challenges frequently triggered regulatory breakdowns, particularly in low-performing groups [16, 26]. High-performing groups, in contrast, demonstrated earlier recognition of emotional strain and more explicit shared regulation of emotions [10, 20]. Consistent with theoretical frameworks emphasizing quality variation in collaborative groups [37], the reviewed literature indicates that positive socioemotional interactions act as a necessary catalyst for achieving a synergy between planning, monitoring, and behavioral engagement.

The literature consistently showed that emotional regulation in collaboration requires shared awareness, interpersonal sensitivity, and coordinated responses to affective cues [5]. Adaptive strategies such as emotion dashboards, reflective prompts, and group awareness tools helped externalise affective states, enabling timely intervention and collective regulation [14, 19]. However, most adaptive systems focused on detecting emotional states rather than supporting sustained group-level emotion regulation. This highlights a need for designs that balance emotional autonomy with shared responsibility, particularly in high stakes learning contexts [5, 20].

### Cognitive regulation

Collaborative learning falters when reasoning processes remain implicit, leading to fragmented problem-solving and shallow consensus [28, 46]. High-performing groups were characterised by explicit articulation of reasoning, shared planning, and coordinated strategy use [28, 46].

Adaptive strategies supporting cognitive regulation focused on externalising thought processes through shared planning templates, dashboards, argumentation tools, and role-based reasoning prompts [14, 48]. These tools supported the development of shared mental models and helped groups negotiate task understanding. However, adaptive systems tended to individualise content difficulty rather than support collective cognitive alignment. The findings suggest that effective adaptive support should be designed to prioritise visibility and synchronisation of reasoning at the group level, rather than solely optimising individual cognitive pathways [14, 48].

### Metacognitive regulation

Metacognitive regulation played a central role in coordinating SRL and SSRL. Across studies, planning, monitoring, and evaluation processes often acted as triggers for transitions from individual regulation to co- and shared regulation [28, 48]. Metacognitive monitoring primarily facilitates this transition, where detecting a gap in group progress serves as a trigger to externalize individual challenges and initiate shared regulation [28, 48]. Groups with aligned monitoring strategies and shared success criteria have demonstrated more stable regulatory trajectories and better learning outcomes.

Misalignment in metacognitive skills, feedback timing, and goal interpretation frequently disrupted collaboration. Adaptive strategies addressing these issues included shared dashboards, group awareness tools, adaptive prompts, and role rotation [14, 47]. These approaches helped make regulation visible and negotiable. However, excessive scripting or poorly timed scaffolds sometimes reduced learner autonomy or interrupted peer regulation. This reinforces the need for adaptive systems that fade support and respond to situational regulatory needs rather than imposing fixed structures [38].

### Motivational regulation

Motivational regulation challenges were closely intertwined with affective and metacognitive processes. Studies reported that misaligned goals, uneven effort, role ambiguity, and lack of shared success structures weakened shared regulation and increased reliance on individual regulatory strategies [7, 11].

While results indicate that adaptive scaffolds show promise for keeping learners engaged, our synthesis revealed critical limitations [19, 47]. Most systems focused on sustaining individual motivation rather than fostering collective motivation. Future studies should design adaptive environments that can support shared ownership, interdependence, and collective efficacy, the key elements of effective collaboration [27, 36].

### Adaptive learning and regulatory integration

Across domains, adaptive learning systems demonstrated strong potential to support regulation through personalised feedback, scaffolding, and analytics. Yet the evidence indicated predominantly individual focus of adaptive designs and a mismatch between the complexity of collaborative regulation [27, 30]. Only a limited number of studies explicitly linked adaptive mechanisms to transitions between SRL, co-regulation, and SSRL [32, 40]. Adaptive systems often responded to regulatory difficulties after breakdowns occurred, rather than proactively supporting the evolving balance between individual and shared regulation [27, 30]. To move beyond reactive support, future adaptive designs should leverage predictive analytics that monitor markers of socio-emotional friction or cognitive misalignment. This would enable proactive scaffolding that facilitates the transition from individual to group regulation before collaboration fails.

This pattern also reflects a conceptual gap. Studies frequently described SRL and SSRL in parallel, but fewer offered integrated explanations of how these processes dynamically interact within adaptive collaborative environments. Without clearer integration, adaptive designs risk reinforcing individualisation at the expense of shared regulation [40].

### Implications for health professions education

Although collaborative competence is central to healthcare practice, few included studies explicitly examined SRL–SSRL dynamics within health professions education, and none provided empirical evidence from medical learners. This gap is notable given the cognitive, emotional, and coordination demands of clinical learning environments [4]. In these settings, the transition to SSRL may be further complicated by power dynamics and professional hierarchies characteristic of clinical teams. These findings support the need for theory-informed adaptive designs in healthcare education that strengthen shared regulation, emotional resilience, and collective decision-making, ensuring that adaptive tools account for the unique high-stakes pressures of the clinical environment.

### Strengths and limitations

This scoping review provides a comprehensive synthesis of literature examining the interaction between self-regulated learning and socially shared regulation of learning within collaborative contexts. By integrating evidence across affective, cognitive, metacognitive, and motivational domains, it advances understanding beyond single-domain or individual-level perspectives that dominate existing research.

Methodological rigour is a key strength. The review followed established scoping review frameworks and reporting guidelines, employed a systematic multi-database search, included selected grey literature, and used independent screening and structured data charting. Hybrid inductive-deductive thematic analysis enabled coherent mapping of regulatory challenges and adaptive strategies across diverse study types. Optional methodological appraisal further strengthened interpretive confidence.

Several limitations should be acknowledged. The review was restricted to English-language publications, which may have excluded relevant international work. The included studies were methodologically and theoretically heterogeneous, limiting direct comparison and precluding quantitative synthesis. Many contributions were conceptual or exploratory, reflecting the early developmental stage of this field. Empirical evidence from health professions education was particularly sparse, limiting context-specific conclusions. In addition, many adaptive strategies were descriptive, with limited evaluation of effectiveness in authentic collaborative settings.

## Conclusion

This scoping review demonstrates that effective collaborative learning depends on a dynamic interplay between self-regulated learning and socially shared regulation across multiple regulatory domains. While adaptive learning environments show promise in supporting regulation, current designs largely prioritise individual-level adaptation and offer limited support for shared regulatory processes. Notably, a critical scarcity of empirical research exists within health professions education. This highlights a major gap for future theory-informed adaptive designs that must harmonise individual autonomy with shared accountability.

## Supplementary Information

Supplementary Material 1.

## Data Availability

All data generated or analysed during this study are included in this published article and its supplementary materials.

## Abbreviations

SRL: Self-regulated learning
SSRL: Socially shared regulation of learning
CSCL: Computer-supported collaborative learning
PRISMA-ScR: Preferred Reporting Items for Systematic Reviews and Meta-Analyses extension for Scoping Reviews
MeSH: Medical Subject Headings
QualSyst: Standard Quality Assessment Criteria for Evaluating Primary Research Papers
MOOC: Massive Open Online Course
