# Supplementary material for: Self-regulated and socially shared regulation of learning in adaptive collaborative educational environments: a scoping review
Source: BMC Med Educ. 2026 Jun 15;26:1360. doi: 10.1186/s12909-026-09355-9 (PMC13501719; doi:10.1186/s12909-026-09355-9)
Supplement: Supplementary file 1 [file 12909_2026_9355_MOESM1_ESM.docx]

Annexure 1: Search Terms Using Keywords, MeSH/Thesaurus Terms, and Free-Text Terms

| Search terms | Search using Boolean operators |
| --- | --- |
| Search terms group 1 | "Self-Control"[MeSH Terms] OR "self-regulat*"[TIAB] OR "Emotional Regulation"[MeSH Terms] |
| Search terms group 2 | "Decision Making, Shared"[MeSH Terms] OR "social* shared regulat*"[TIAB] OR "collaborat* learn*"[TIAB] OR "group learn*"[TIAB] OR "team learn*"[TIAB] OR "social learn*"[TIAB] OR "co-regulat*"[TIAB] OR "shared regulat*"[TIAB] OR "collective learn*"[TIAB]) |
| Search terms group 3 | "adapt* learn*"[TIAB] OR "adapt* learn* environment*"[TIAB]  OR "personaliz* learn*"[TIAB] OR "individualiz* learn*"[TIAB]  OR "adapt* education"[TIAB] OR "intelligent tutor*"[TIAB] |
| Search terms group 4 | "Learning"[MeSH Terms] OR "learn*"[TIAB] OR "Teaching"[MeSH Terms] OR "Education"[MeSH Terms] OR "Models, Educational"[MeSH Terms] OR "medical education"[TIAB] OR "higher education"[TIAB] |
| Search strategy  (search terms “group 1” AND “2” AND “3” AND “4”) | ("Self-Control"[MeSH Terms] OR "self-regulat*"[TIAB] OR "Emotional Regulation"[MeSH Terms] )  **AND**  ("Decision Making, Shared"[MeSH Terms] OR "social* shared regulat*"[TIAB] OR "collaborat* learn*"[TIAB] OR "group learn*"[TIAB] OR "team learn*"[TIAB] OR "social learn*"[TIAB] OR "co-regulat*"[TIAB] OR "shared regulat*"[TIAB] OR "collective learn*"[TIAB])  **AND**  ("adapt* learn*"[TIAB] OR "adapt* learn* environment*"[TIAB]  OR "personaliz* learn*"[TIAB] OR "individualiz* learn*"[TIAB]  OR "adapt* education"[TIAB] OR "digital learn*"[TIAB] OR "intelligent tutor*"[TIAB])  **AND**  ("Learning"[MeSH Terms] OR "learn*"[TIAB] OR "Teaching"[MeSH Terms] OR "Education"[MeSH Terms] OR "Models, Educational"[MeSH Terms] OR "medical education"[TIAB] OR "higher education"[TIAB]) |

Annexure 2: Data Extraction Template Used for Included Studies

| **Field** | **Description / Data to Extract** |
| --- | --- |
| **Author(s) & Year** | Full citation used for identification |
| **Study Type** | Conceptual, empirical (quantitative/qualitative/mixed-methods), systematic review, theoretical model paper. |
| **Study Setting / Context** | Educational level, discipline, and setting (e.g., higher education, healthcare education, digital learning environment). |
| **Objective / Purpose of Study** | Brief statement summarizing the main aim or research question. |
| **Key Concepts Addressed** | SRL, SSRL, co-regulation, collaborative learning, adaptive learning, or theoretical model evaluated. |
| **Major Findings / Key Contribution** | Summary of the study’s contribution, key results, or conceptual insight relevant to regulatory learning. |
| **Emergent Theme (From This Review)** | Theme assigned during thematic analysis (e.g., metacognitive awareness, adaptive support, SRL–SSRL interaction). |

**Annexure 3: Characteristics of Included Studies (n=31)**

| Sr No. | Title | Author(s) & Year | Study Type | Study Setting / Context | Objective / Purpose of Study | Key Concepts Addressed | Emergent Theme (From This Review) |
| --- | --- | --- | --- | --- | --- | --- | --- |
|  |  |  | **QualSyst Score (if applicable)** |  |  |  |  |
| 1. | Self-regulation and shared regulation in collaborative learning in adaptive digital learning environments: A systematic review of empirical studies | (Sharma et al., 2024) | Systematic literature review of empirical studies. | Adaptive digital learning environments; individual and collaborative learning contexts. | To review empirical evidence on adaptive learning environments that foster self-regulation and shared regulation of learning in collaborative settings. | Self-regulation (SRL), socially shared regulation of learning (SSRL), adaptive learning technologies, feedback, scaffolding, learning trajectories, collaborative learning processes, self-assessment, help-seeking behaviour. | Adaptive technologies to promote self-regulated and shared regulation of learning in collaborative contexts. |
| 2. | A Review of Self-regulated Learning: Six Models and Four Directions for Research | (Panadero, 2017) | Conceptual / theoretical review of six major SRL models | Educational psychology; SRL research across student developmental levels and educational stages | To analyze, compare, and synthesize six major SRL models and propose future research directions | SRL, metacognition, cognition, motivation, emotion, volition, SRL models (Zimmerman, Boekaerts, Winne & Hadwin, Pintrich, Efklides, Hadwin–Järvelä–Miller) | Theoretical foundations of SRL; model comparison; developmental differences in SRL; framework integration |
| 3. | Self-Regulation, Coregulation, and Socially Shared Regulation: Exploring Regulated Learning Theory | (Hadwin & Oshige, 2011) | Conceptual / Theoretical Review | To introduce and contrast social aspects across three perspectives: self-regulated learning (SRL), coregulated learning (CoRL), and socially shared regulation of learning (SSRL). | To examine whether previous performance and motivational antecedents (self-efficacy, task values) predict self-regulation, co-regulation, and socially shared regulation of intrinsic motivation, and to compare differences between university students and working adults. | Theoretical positioning of "social" in SRL; operational definitions of SRL, CoRL, and SSRL; multimethodological approaches | Theoretical foundations and conceptual boundaries of social regulation. |
| 4. | Socially Shared Regulation of Learning in CSCL: Understanding and Prompting Individual- and Group-Level Shared Regulatory Activities | (Järvelä, Kirschner, et al., 2016) | Conceptual / Theoretical Review | Computer-Supported Collaborative Learning (CSCL) environments | To review prior CSCL support lines and discuss how SSRL contributes to effective CSCL, what tools exist, and future directions for tool development. | SSRL, self-regulation, co-regulation, collaborative learning, CSCL support tools, cognitive–motivational–emotional–social regulation. | Role of metacognitive and shared regulatory support in CSCL |
| 5. | Socially Shared Regulation of Learning and Participation in Social Interaction in Collaborative Learning | (Isohätälä et al., 2017) | Qualitative empirical study (video-based micro-analytic observation) | 2nd-year teacher-education students in a mathematics education course at a Finnish university; small-group collaborative tasks; six groups video-recorded across two lessons. | To investigate how SSRL emerges during fluctuations in students’ participation in social interaction during collaborative learning. | SSRL, participation patterns, collaborative learning interaction, micro-analytic coding, situative challenges, coordination. | Fostering socially shared regulation through active social interaction. |
|  |  |  | 95% |  |  |  |  |
| 6. | Putting Self-Regulated Learning in Context: Integrating Self-, Co-, and Socially Shared Regulation of Learning | (Bransen et al., 2022) | Conceptual / Theoretical paper | Healthcare education and collaborative healthcare practice; discussion situated within health professions education and higher education | To examine how existing conceptualizations of regulatory learning (SRL, CoRL, SSRL) align with the growing need for collaboration and collective competence in healthcare, and to propose integrated ways of conceptualizing regulated learning in healthcare teams | Self-regulated learning (SRL); Co-regulation (CoRL); Socially shared regulation of learning (SSRL); Collaborative learning; Collective competence; Regulation in healthcare education | Importance of SRL–CoRL–SSRL integration for collaborative learning in healthcare settings |
| 7. | Online Adaptive Learning: A Study of Score Validity of the Adaptive Self-Regulated Learning | (Harati, 2019) | Quantitative validation study (instrument development: reliability + validity testing) | Adaptive online learning environment (ALEKS), university chemistry course, U.S. | To design and empirically evaluate a theoretical model of Adaptive Self-Regulated Learning (ASR) and validate the new Adaptive Self-Regulated Learning Questionnaire (ASRQ). | Adaptive Learning (AL), Self-Regulated Learning (SRL), Adaptive SRL model, instrument development, reliability, validity, confirmatory factor analysis. | Adaptive SRL measurement; structural components of SRL in adaptive digital environments; validation of SRL tools for ALE-based learning. |
|  |  |  | 95.45% |  |  |  |  |
| 8. | Promoting Socially Shared Regulation of Learning in CSCL: Progress of Socially Shared Regulation Among High- and Low-Performing Groups | (Malmberg et al., 2015) | Qualitative process-oriented study (content analysis + process discovery) | Teacher-education multimedia course; two-month duration; CSCL environment using the VCRI platform; groups of 3–4 students (N = 103) | To explore how collaborative groups progress in their socially shared regulation of learning (SSRL) during CSCL, and how high- and low-performing groups differ in their SSRL focus and progression | SSRL, collaborative learning, CSCL, regulation strategies, challenges in collaboration, group performance differences | SSRL progression varies by group performance; effective SSRL requires adaptive shifts in strategy focus over time |
|  |  |  | 78% |  |  |  |  |
|  |  |  |  |  |  |  |  |
| 9. | Adaptive and Intelligent Systems for Collaborative Learning Support: A Review of the Field | (Magnisalis et al., 2011) | Structured literature review / systematic review of 105 studies (70 with evaluation data). | Global published research on adaptive and intelligent systems for collaborative learning support (AICLS), including AI-based and Web-2.0 supported collaborative learning environments. | To analyze critical design issues of AICLS systems, propose a unified classification scheme, evaluate evidence of learning impact, and identify emerging trends and open research questions. | Adaptive collaborative learning, AI-supported learning systems, peer interaction support, modeling approaches, pedagogical objectives, adaptation targets, system design principles, pre-task and in-task interventions. | AI-mediated collaborative learning; adaptive support for regulation and interaction; design principles for technology-supported collaborative environments. |
| 10. | Different Futures of Adaptive Collaborative Learning Support | (Rummel et al., 2016) | Conceptual / Theoretical Position Paper | Future-oriented discussion in Computer-Supported Collaborative Learning (CSCL) and Adaptive Collaborative Learning Support (ACLS) research communities. | To contrast dystopian vs. utopian futures for adaptive collaborative learning support and outline a 25-year research agenda for theory-driven, nuanced, and transparent ACLS design | Adaptive collaborative learning support (ACLS), CSCL, AI-augmented collaboration, instructional frameworks, dimensions of support (timing, mode, realm, locus, type), adaptivity vs. adaptability | Role of AI in shaping future ACLS; need for theory-grounded adaptive support; risks of poorly aligned automation; emphasis on learner autonomy and transparency |
| 11. | Capturing Regulatory Patterns in Online Collaborative Learning: A Network Analytic Approach | (Zhang et al., 2021) | Mixed-methods (content analysis + ENA) | 45 student teachers in 8-week online collaborative learning activities | To trace dynamic regulatory patterns across stages of online collaborative learning and compare high- vs low-performing groups | SSRL, content monitoring, evaluating, social-emotional regulation, epistemic network analysis (ENA) | Cognitive–social regulation patterns predicting group performance |
|  |  |  | 71% |  |  |  |  |
| 12. | Capturing Temporal and Sequential Patterns of Self-, Co-, and Socially Shared Regulation in the Context of Collaborative Learning | (Malmberg et al., 2017) | Mixed-Methods (Qualitative + Lag Sequential Analysis) | Two-month math didactics course; teacher-education students working in 3-member collaborative groups; 22 hours of video recorded across 6 groups | To examine temporal and sequential patterns of self-, co-, and socially shared regulation events during different stages of collaborative learning | Self-regulation (SR), Co-regulation (CoRL), Socially Shared Regulation (SSRL); metacognitive monitoring; planning; task execution; temporal sequences | Temporal Dynamics of SSRL—task execution acts as a trigger for shared planning; monitoring drives forward progression |
|  |  |  | 75% |  |  |  |  |
| 13. | Capturing the Dynamic and Cyclical Nature of Regulation: Methodological Progress in Understanding Socially Shared Regulation in Learning | (Järvelä et al., 2019) | Conceptual / Methodological Review | Review of SSRL methodological research in collaborative and CSCL learning contexts | To review methodological progress in capturing temporal, sequential, and situative characteristics of SSRL in collaborative learning | SSRL; SRL; co-regulation; trace data; sequential/temporal analysis; multimodal data; technological tools; in-situ regulation; challenges triggering regulation | Methodological advancements for capturing SSRL dynamics |
| 14. | Enhancing Socially Shared Regulation in Collaborative Learning Groups: Designing for CSCL Regulation Tools | (Järvelä et al., 2015) | Conceptual / Design Framework paper | No empirical sample; theoretical analysis with illustrative CSCL tools (Radar, OurPlanner) | To propose design principles for supporting socially shared regulation in CSCL environments and demonstrate how technological tools can scaffold SSRL | Socially Shared Regulation of Learning (SSRL); group awareness; externalization tools; planning and monitoring support; CSCL affordances | Conceptual foundations for SSRL-support design; guidance for building adaptive tools that scaffold shared planning, monitoring, and strategy use |
| 15. | Exploring College English Language Learners’ Self- and Social Regulation of Learning During Wiki-Supported Collaborative Reading Activities | (Lee et al., 2021) | Mixed-methods (Content analysis + Sequential analysis) | Chinese university; 60 EFL college students; 12 groups; 16-week wiki-supported collaborative reading tasks | To examine how EFL learners use self- and social regulation during wiki-supported collaborative reading, and how regulatory patterns differ between high- and low-performing groups | Self-regulation, co-regulation, socially shared regulation; regulation type, process, focus; CSCL; wiki-based collaboration | SSRL in language-learning contexts; performance differences linked to depth and fluidity of regulation sequences |
|  |  |  | 64% |  |  |  |  |
| 16. | Fostering Regulation of Learning Processes Among Programming Students Using Computational Scaffolding | (Mendes et al., 2023) | Randomized Controlled Trial (single-blind RCT) | Online introductory programming course; students enrolled in a CS-related program; collaborative learning environment with computational scaffolding features. | To test whether different types of computational scaffolding (SRL-only vs. SRL+Co-Reg+SSRL vs. no support) improve regulation of learning and course performance in collaborative programming tasks. | Self-regulated learning (SRL); co-regulation; socially shared regulation of learning (SSRL); computational scaffolding; collaborative learning; online programming education. | Impact of SSRL-specific scaffolds on collaborative task performance |
|  |  |  | 89.3% |  |  |  |  |
| 17. | New Frontiers: Regulating Learning in CSCL | (Järvelä & Hadwin, 2013) | Conceptual / theoretical review | CSCL context (no empirical setting) | Explains SRL, co-regulation, SSRL; links regulation theory with CSCL tools; outlines how CSCL can support cognitive, motivational, and socio-emotional regulation | Collaboration requires SRL, co-regulation, SSRL; individual SRL tools can extend to groups; CSCL knowledge-building tools can scaffold regulation; effective collaboration needs shared awareness, joint monitoring, and coordinated strategies | Not applicable (non-empirical paper) |
| 18. | Recognizing Socially Shared Regulation by Using Temporal Sequences of Online Chat and Logs in CSCL | (Järvelä, Malmberg, et al., 2016) | Mixed-methods (quantitative log-trace + qualitative content analysis) | Graduate-level CSCL course using nStudy platform; 5 groups over 3 collaborative phases | To examine temporal sequences of self- and socially shared regulation during collaborative learning and their link with learning outcomes | Self-regulated learning events; socially shared regulation episodes; chat-based interaction; log-trace activities; temporal sequencing; learning outcomes (SOLO-based ratings) | Shared task understanding; shared planning; shared strategy use; shared motivation; SRL phases (forethought, performance, reflection) |
|  |  |  | 78.6% |  |  |  |  |
| 19. | Supporting Groups’ Emotion and Motivation Regulation During Collaborative Learning | (Järvenoja et al., 2017) | Mixed-methods (tool-generated quantitative states + qualitative video-coded regulation episodes) | Higher-education math-didactics course; 44 university students; 11 groups; 41 recorded collaborative sessions | To examine when and how co- and socially shared emotion and motivation regulation occur during collaboration and whether the S-REG tool supports these processes | Emotional state; motivational state; traffic-light S-REG values; co-regulation episodes; shared-regulation episodes; duration patterns; session-phase patterns | Socio-emotional challenges; transitions from co- to shared regulation; early-session vulnerability; group-level awareness via S-REG; motivational dips and recovery |
|  |  |  | 67.8% |  |  |  |  |
| 20. | Understanding Regulated Learning in Situative and Contextual Framework | (Järvenoja et al., 2015) | Non-empirical (conceptual) | Theoretical paper with an illustrative video-based case example | To explain regulated learning through a situative, context-sensitive framework; to describe multilayered regulation in social learning | Situative regulation; contextual features; interaction records; multilayer analysis; socially shared regulation; emotion–motivation dynamics | Context dependence; interaction-driven meaning-making; dynamic shifts in emotions/motivation; multilayer observation; blending individual and shared regulation |
| 21. | Promoting regulation of equal participation in online collaboration by combining a group awareness tool and adaptive prompts. But does it even matter?ollaboration by combining a group awareness tool and adaptive prompts. But does it even matter? | (Strauß & Rummel, 2021) | Mixed-methods experimental field study | University online MOOC-style course; 16–13 groups; two 2-week collaborative tasks | To test whether a group awareness tool and adaptive prompts improve equal participation in online collaboration | GAT; adaptive prompts; participation distribution; Gini coefficient; coordination sequences; satisfaction; self- and socially shared regulation | Unequal workload; limited use of external regulation tools; value of early coordination; dependency on group habits rather than prompts |
|  |  |  | 71% |  |  |  |  |
| 22. | More Socio-Emotional Regulation: Exploring Social Regulation of Learning in Collaborative Argumentation Among High and Low Performing Groups | (Li et al., 2023) | Qualitative | Higher-education groups; 90-min collaborative argumentation task; 14 groups, high vs low performers | To compare high- and low-performing groups in challenges, social regulation, and sequential regulation patterns | Socio-emotional challenges; cognitive challenges; motivational challenges; social regulation sequences; argument diagram scores | Tense group climate; deadlock resolution; uneven attention to member participation; different socio-emotional trajectories |
|  |  |  | 90% |  |  |  |  |
| 23. | Emotion Control in Collaborative Learning Situations: Do Students Regulate Emotions Evoked by Social Challenges? | (Järvenoja & Järvelä, 2009) | Mixed-methods empirical | University teacher-education course; groups of 3–5 students (n=63); three collaborative learning tasks | To examine socio-emotional challenges in collaboration and the use of self-, other-, and shared-regulation of emotions | Socio-emotional challenges; self-, other-, shared-emotion regulation; group dynamics; collaborative tasks | Differences across tasks; interaction between personal goals and group functioning; variation in regulation types; socially triggered emotional episodes |
|  |  |  | 91% |  |  |  |  |
| 24. | Emotional Regulation in Collaborative Learning: When Do Higher Education Students Activate Group-Level Regulation in the Face of Challenges? | (Järvenoja et al., 2019) | Mixed-methods empirical | Higher-education mathematics course; 15 groups; 3–4 students per group; 6 collaborative tasks; 87 h video | To identify challenges that activate group-level emotion regulation and to classify co- and socially shared regulation strategies | Cognitive, emotional, motivational, and interaction challenges; co-regulation; socially shared regulation; regulation strategies | Dynamic emergence of regulation; interplay between challenge type and strategy use; fine-grained temporal analysis |
|  |  |  | 91% |  |  |  |  |
| 25. | Regulation During Cooperative and Collaborative Learning: A Theory-Based Review of Terms and Concepts | (Schoor et al., 2015) | Non-empirical conceptual/theoretical review | Literature on cooperative and collaborative learning | To clarify how regulation terms are used; to categorize regulation modes using a feedback-loop model | Self-regulation; co-regulation; socially shared regulation; other-regulation; regulatory feedback loop; cooperative vs collaborative learning | Conceptual confusion; overlap between modes of regulation; need for shared definitions; aligning terminology with Winne & Hadwin model |
| 26. | A Framework for Adaptive Learning Design in a Web-Conferencing Environment | (Bower, 2015) | Qualitative; Design-Based Research | Three semesters of online graduate programming classes using Adobe Connect | To develop a framework for adaptive learning design in synchronous web-conferencing environments | Adaptive learning design; dynamic interface redesign; collaboration; multimodal interaction; DBR cycles | Importance of spontaneity in design changes; teacher as participant-researcher; multimodal load; role of failures in shaping redesign principles |
|  |  |  | 80% |  |  |  |  |
| 27. | Systematic Review of Adaptive Learning Research Designs, Context, Strategies, and Technologies from 2009 to 2018 | (Martin et al., 2020) | Systematic Review (Non-empirical) | Review of 61 studies (2009–2018) | To synthesize research trends, contexts, methods, adaptive strategies, and technologies in adaptive learning | Publication patterns; instructional contexts; research design components; adaptive strategies; adaptive technologies | Gaps in methodological diversity; heavy reliance on learning-style models; limited exploration of advanced adaptivity; need for more research on emerging adaptive technologies |
| 28. | Reviewing Findings on Socially Shared Regulation of Learning | (Panadero & Järvelä, 2015) | Narrative Review (Non-empirical) | Review of 17 studies | To examine the empirical evidence for SSRL as a distinct phenomenon from co-regulation and to identify how it is typically researched. | Confirmed the existence of SSRL as a separate construct. It noted that the field relies heavily on mixed methods and video-recorded observation, and that SSRL generally correlates with better performance. | Identifying group characteristics and optimal conditions for SSRL |
| 29. | Research Landscape of Adaptive Learning in Education: A Bibliometric Study on Research Publications from 2000 to 2022 | (Jing et al., 2023) | Non-empirical; Bibliometric analysis | WoS-indexed 644 papers (2000–2022) | To map publication trends, research evolution, clusters, and frontiers in adaptive learning | Publication volume; authorship networks; national output; journal sources; research clusters; keyword bursts | Rapid field growth; technology-driven evolution; dominance of computer science journals; shift toward deep learning & knowledge tracing; need for cross-disciplinary expansion |
| 30. | A Framework for Self-Regulated Digital Learning (SRDL) | (Yen et al., 2018) | Conceptual / Theoretical | Not applicable (framework synthesis) | To develop a comprehensive framework for self-regulated digital learning (SRDL) in e-learning environments | SRDL features: learning plan; e-portfolio; evaluation; human feedback; machine feedback; visualization; scaffolding; agents; SRL skills (planning, monitoring, evaluating, cognitive strategies) | Need for SRL-embedded digital environments; gaps in holistic SRL support; importance of combining human feedback, AI feedback, and scaffolds |
| 31. | Paradigmatic Dimensions of Instrumentation and Analytic Methods in Research on Self-Regulated Learning | (Winne, 2019) | Conceptual / Theoretical | Higher Education / Learning Sciences / CSCL Research Environments | To examine the relationship between instrumentation (data-gathering tools), analytic methods, and the underlying theoretical paradigms that shape how Self-Regulated Learning (SRL) is measured and understood. | 4-Phase Model of SRL (Environmental Scanning, Goal Setting, Strategic Enactment, Adaptation) | Need for multimodal adaptive tools to capture the non-linear, adaptive phases of regulation that traditional surveys "pass over." |

**Annexure 4: Evidence mapping of thematic analysis**

| Regulatory Challenges | Adaptive Strategies |
| --- | --- |
| Affective/Emotional Domain | |
| Emotional Autonomy vs. Group Influence  • Peer feedback delivered with poor timing or tone  • Group stress dominating individual emotion management  • Overbearing social correction or judgment | **Structured Emotional Regulation in Collaborative Settings**  • Use emotion dashboards to track team affect  • Rotate roles to distribute emotional labour  • Implement self-monitoring routines and peer check-ins |
| Emotional Disconnect in Group Dynamics  • Lack of attunement/social grounding  • Emotional disconnect  • Invisible affective states  • Unspoken frustrations or discomfort | **Socioemotional Awareness & Climate Tools**  • Emoji check-ins and sentiment analysis tools  • Emotion-sharing widgets embedded in CSCL platforms  • Reflective journaling with peer visibility access  • Norm-setting for emotional respect |
| Unregulated Affective Traits  • Anxiety-prone learners underperform  • Emotionally dominant individuals overshadow peers  • No mechanism for managing group affective balance | **Emotionally Adaptive Group Structuring**  • Pre-task emotional profiling  • Mood-tracking tools for adaptive grouping  • Individualized support scaffolds based on affective profiles |
| Behavioural Style Clashes in Collaboration  • One member dominates; others disengage  • Fast vs. slow pacing disrupts cohesion  • Differing expectations for response time/structure | **External Behavioural Scaffolds**  • Pacing guides and shared task trackers  • Role-based protocols and turn-taking scripts  • Group contracts for behavioural expectations |
| Unequal Engagement & Task Ownership  • Passive participants relying on emotionally driven leaders  • Burnout in highly committed members  • Resentment due to perceived inequity | **Equity-Based Behavioural Scaffolding**  • Rotating task ownership roles  • Visual contribution dashboards  • Pair-sharing and mutual accountability tools |
| Environmental & Attention Regulation Gaps  • Distracting environments with no attention cues  • Uneven task structuring causes frustration  • Misalignment of external stimuli disrupts regulation | **Task & Attention Management Tools**  • Group checklists and task planners  • Digital attention prompts and timers  • Use of planners, goal sheets, timers, and environmental control tools |
| Cognitive Domain | |
| Prior Knowledge & Cognitive Diversity Mismatch  • Mismatch in domain knowledge  • Misaligned learning strategies  • Diverse thinking styles  • Conflicting mental models | **Adaptive Group Formation & Matching• Group formation based on learning profiles• Peer matching by style/knowledge• Adaptive profiling tools• Jigsaw & adaptive tutoring approaches** |
| Isolation from Over-Personalized Learning  • Isolation due to modality adaptation  • Lack of interactive content  • Divergence in cognitive processing | **Modality & Content Adaptation for Cognitive Balance**  • Dynamic multimodal content delivery  • Group-based instructional sequences  • Pre-class differentiated content prompts |
| Alignment & Visibility of Cognitive Processing  • Lack of shared strategies  • Uncoordinated problem solving  • Invisible cognitive decision-making | **Harmonization & Visualization of Cognitive Strategies**  • Collaborative planning tools  • Strategy boards and PBL scaffolds  • Guiding/mirroring tools and dashboards |
| Disruptive Learning Design  • Technological mismatches  • Excessive rigidity in task structure  • Poor engagement with content flow | Cognitive Flexibility Tools• Redesign atomic tasks for flexibility• Flexible scripting tools• Collaborative checkpoints |
| Cognitive Load & Autonomy–Collaboration Balance  • High cognitive load  • Executive function overload  • Fragmented autonomy | **Task Complexity Matching & Chunking**  **•** Scaffolded chunking methods  • Task-complexity balancing tools  • Balanced individual/group roles and expectations |
| Metacognitive Domain | |
| Misaligned Monitoring & Regulation Skills  • Varied monitoring skills  • Difficulty integrating SRL, co-, and SSRL  • Unsynced feedback loops  • Misaligned goals | **Shared Monitoring & Visualization Tools**  • Shared dashboards  • Group awareness tools  • Team-synced e-portfolios  • Visualization tools |
| Overscripting vs. Underscripting  • Overscripting reduces SRL  • Fixed scripts stifle skills  • Misaligned scripting disrupts monitoring | **Adaptive Scripting & Support Fading**  **•** Adaptive scripts vs. Fixed  • LMS conditional logic  • Adaptive turn-taking protocols  • Script fading |
| Misaligned Feedback & Scaffolds  • Directive or mistimed scaffolds  • Over-reliance on teacher feedback | **Feedback for Regulation Adaptation**  **•** Context-aware feedback  • Group monitoring & alerts  • Intelligent agents nudging strategy shifts |
| Goal Misalignment  • Divergent individual/group goals  • Divergent e-portfolio tracking | **Goal Alignment & Tracking Systems**  • Shared tracking systems  • Goal alignment check-ins  • Personal feedback integrated into group metrics |
| Inflexible Peer Interaction Models  • Peer models lack adaptation  • Missed trigger events  • No structured reflection | **Reflection & Debriefing Integration**  **•** Debriefing in simulations  • Peer reflection prompts  • Shared journals |
| Motivational Domain | |
| Goal Misalignment in Motivation  • Failure to align individual and group goals  • Achievement vs. competence conflicts  • Self-set goals vs. team expectations  • Mastery vs. performance-avoidance tension | **Goal Alignment & Motivation Mapping**  • Narrative constructor tools  • Shared goal setting and feedback loops  • Align group goals with personal standards |
| Variability in Engagement & Effort  • Inconsistent motivational states  • Mismatch in effort levels  • Unequal responsibility | **Adaptive Feedback & Nudging Systems**  • Real-time motivation feedback  • Personalized dashboards and nudges  • Competence-oriented feedback loops |
| Role Ambiguity  • Unclear collaborative roles  • Unequal contribution  • Confused expectations | **Role-Based Motivation Design**  • Rotating collaborative roles  • Scaffolds for social contribution  • Co-constructed interdependent roles |
| Autonomy vs. External Motivation Control  • Over-scaffolding reduces autonomy  • Group pressure conflicts with self-determination  • Extrinsic motivation clashes | **Positive Interdependence Frameworks**  • Positive interdependence mechanisms  • Shared outcomes and collective rewards  • Interdependence embedded in system architecture |
| Lack of Shared Success Structures  • Missing interdependence demotivates  • AI-driven systems reduce equity  • No shared reward logic | **Motivation-Driven Environment Design**  • Balance tech with human feedback  • Prevent inequality through motivational design  • Autonomy-supportive prompts and challenges |
